# Trends in adolescent mental health problems 2004–2020: Do sex and socioeconomic status play any role?

**DOI:** 10.1177/14034948231165552

**Published:** 2023-05-04

**Authors:** Benti Geleta Buli, Peter Larm, Kent Nilsson, Fabrizia Giannotta

**Affiliations:** 1 Department of Public Health Sciences, Mälardalen University, Västerås, Sweden; 2 Department of Public Health Sciences, Stockholm University, Stockholm, Sweden; 3 Center for Clinical Research, Uppsala University, Västmanland County Hospital, Västerås, Sweden; 4 Department of Neuroscience, Uppsala University, Uppsala, Sweden

**Keywords:** Trends, mental health, adolescents, sex, socioeconomic status, SES

## Abstract

**Aims:**

This study aims to investigate trends in four types of adolescent mental health problems; that is, psychosomatic symptoms, depressive symptoms, suicidal ideations, and suicide attempts 2004–2020. A second aim is to investigate the moderating roles of socioeconomic status and sex in these trends.

**Methods:**

The analysis is based on repeated cross-sectional data 2004–2020 among grade 9 students in secondary schools in a Swedish county. In total, data from 19,873 students were included in the analysis. We fitted linear and logistic regression equations and used survey-years’ coefficients to estimate the trends. We also estimated the moderating effects of socioeconomic status and sex using interactions between survey year and socioeconomic status and sex, respectively.

**Results:**

The trends in all mental health problems declined over time. Through its interaction with survey year, socioeconomic status moderated the trends; psychosomatic symptoms (B = −0.115, *P*<0.001), depressive symptoms (B = −0.084, *P*<0.001) and suicidal ideations (odds ratio 0.953, confidence interval 0.924–0.983) significantly declined over time among those with high socioeconomic status. However, socioeconomic status did not have an association with the trend in suicide attempts. Interaction between sex and year of survey was associated with significant decreasing trends in depressive symptoms and suicidal ideations only among girls.

**Conclusions:**

**Adolescent mental health problems have decreased over time, but only for adolescents with high socioeconomic status, or only in depressive symptoms and suicidal ideations for girls. The results shed light on the growing inequalities in health outcomes across levels of socioeconomic status.**

## Background

There is considerable evidence that adolescent mental health problems are on the rise. Several studies have shown a significant increase in recent decades [1–3]. This poses public health concerns, because about half of all mental health problems in adults begin before or during adolescence [4].

Previous studies that have shown increased trends in mental health problems have often focused on one or a few dimensions of mental health. For instance, some have used psychosomatic symptoms (PSSs) to estimate trends in mental health problems among adolescents [5, 6]. Others have included depressive symptoms (DSs) [1, 7], but only a few have incorporated suicide rates [2, 8]. Mental health problems are complex and comprise multiple domains of problems of varying severity [9, 10] that may be influenced differently by various factors. Hence, despite high comorbidity between the domains [11], they may have varying trends [12]. Therefore, studying multiple problems of varying degrees of severity may provide a more comprehensive picture of the trends in youth mental health [10, 13].

To our knowledge, there are a few studies [1, 2, 11] that have focused on multiple mental health conditions rather than just a few aspects, such as PSSs or psychological distress. Blomqvist et al. [1] used data collected at only two time points, 1981 and 2014, although more than two are recommended to analyse time-specific factors for trends in adolescent mental health [5]. Bremberg [2], on the other hand, covered the period 1990–2010 and that raises the need to explore the situation in more recent years. Weinberg et al. [11] was the only study we found to have used multiple indicators as well as data as recent as 2017, indicating the need for more studies of this kind. The present study bridges the gaps in research by analysing more indicators using more recent data collected through repeated surveys.

Another limitation of the literature lies in the general scarcity of studies regarding the role of socioeconomic status (SES) in the trends in mental health. The increasing socioeconomic inequalities in health among adolescents in Sweden [14] necessitate investigation of whether these inequalities are related to trends in mental health problems. Among the few studies that have shown such associations are those by Kim et al. [15], Kim and Hagquist [5] and Weinberg et al. [11]. Like our study, Kim et al. [15] focused on subjective SES while the other two studies combined both subjective and objective SES. Subjective SES refers to people’s perceptions and experiences of their socioeconomic ranking by averaging the standard markers of SES, including education status, occupation, household assets, income, and other markers [16]. Of these three studies, only Kim et al. [15] presented findings consistent with previous studies that found strong associations between subjective SES and psychological functioning and ill health [17, 18]. Although Kim and Hagquist [5] found a negative influence of low family SES on adolescents’ mental health, the study focused on family situations during times of economic difficulties, and may not be generalisable to any ordinary period. Given the scarce evidence in this regard, more studies are needed to investigate the impact of SES on mental health trends.

## Aims

In this study, we aimed to analyse trends in PSSs, DSs, suicidal ideations (SIs) and suicide attempts (SAs) in Swedish adolescents aged 15 years and examined the moderating role of SES. Moreover, as many studies have indicated that girls’ mental health has declined more than boys’ over time; for example, Campbell et al. [19] and Högberg et al. [20], we investigated the role of sex in mental health trends.

## Methods

### Participants

We used data from the Survey of Adolescent Life in Västmanland (SALVe), a project that has collected data every 2–3 years since 1995, to monitor the psychosocial health of adolescents in Sweden’s Västmanland region. Surveys were conducted in all secondary and upper secondary schools in the county. Students in special schools and those with insufficient Swedish language skills were excluded. All the surveys were paper based except in 2020, when the survey was conducted online. The questionnaires were completed during class hours under supervision of teachers and took about 1 hour to complete. The current study was based on data collected from a total of 19,873 students in grade 9 (15-year-olds) in all schools, except special schools, in the county in 2004–2020. The overall average response rate was 80.4%, with the lowest (75%) in 2017 and the highest (87%) in 2014. Boys and girls equally constituted the sample, and about 84.8% of the participants had high SES. However, data were not uniformly available throughout the study period (2004–2020) for all the mental health domains in this study (see Table I). For example, information specific to DSs and SIs was collected until 2012, while that specific to PSSs was collected until 2014. On the other hand, data on SAs were available for all the waves of data collection except in 2014. Hence, the sample sizes varied. Given the small proportions of missing data, which ranged from zero for the year-of-survey variable to 9% for SAs, we used listwise deletion to deal with missing values. As a result, *n*=15,127 for PSSs, *n*=13,138 for DSs, *n*=12,659 for SIs, and *n*=15,397 for SAs were included in the analyses (see Supplemental Table IV). Västmanland’s population is considered fairly representative of the country, based on the distributions of education, employment and income levels as well as urban and rural settings [21]. As the surveys were completely anonymous, application for ethical approval was not required according to Swedish law (Ethical Review Act 2003: 460).

**Table I.** Total number of participants by survey year, type of dependent variable included, sex, and SES.

Year of survey	Dependent variables included	Total	Girls (%)	High SES (%)	Response (%)^a^
2004	PSSs, DSs, SIs, SAs	2899	49.5	79.6	80.0 (a)
2006	PSSs, DSs, SIs, SAs	3207	49.2	82.8	80.3 (b)
2008	PSSs, DSs, SIs, SAs	2690	49.4	85.4	78.2 (c)
2010	PSSs, DSs, SIs, Sas	2622	49.7	84.8	82.7 (d)
2012	PSSs, DSs, SIs, SAs	2121	49.2	86.4	82.0 (e)
2014	PSSs	2211	50.9	87.6	87.0 (f)
2017	SAs	2006	50.8	86.4	75.0 (g)
2020	SAs	2117	52.3	89.0	77.8 (h)
Total		19 873	50.0	84.8	80.4

PSSs: psychosomatic symptoms; DSs: depressive symptoms; SIs: suicidal ideations; SAs: suicide attempts; SES: subjective social status and subjective economic wealth.

a Sources of response rates information: (a) Åslund et al., 2007; (b) Åslund et al., 2009; (c) Hellström et al., 2015; (d) Västmanland regional report, 2010; (e) Kerstis et al., 2012; (f) Västmanland regional report, 2014; (g) Institute for Quality Indicators in Västmanland, 2017; (h) Västmanland regional report, 2020.

### Instruments

#### Psychosomatic symptoms

To measure PSSs, we used a scale constructed through summation of the participants’ responses to questions about the following eight items: headache, stomach ache, pain in the shoulders or neck, pain in the back or hips, pain in hands/knees/legs/feet, difficulty in sleeping, feeling nervous, and feeling irritated. The participants were asked if they had experienced any of the eight symptoms during the 3 months prior to the date of data collection. The response categories for each item/question ranged from never (0) to always (4). A dependent index variable was created from this summation with the total score ranging from 0 to 32 points (Cronbach’s α = 0.81, range 0.80–0.82 across the waves) in which higher scores indicate worse psychosomatic health. The use of such a scale was reported in a previous study, see Hagquist [6].

#### Depressive symptoms

The adolescent version of the Depression Self-Rating Scale (DSRS-A) was employed, in which 15 single items were transformed into nine different symptoms based on the criteria for major depression in the fourth edition of the Diagnostic and Statistical Manual of Mental Disorders (DSM-IV) [22]. In the surveys, the adolescents were asked if they had experienced any of the DSs during the 2 weeks prior to the date of data collection. They responded ‘yes’ (1) or ‘no’ (0) to each question. An index variable (α = 0.85, range 0.83–0.86 across the waves) was created from summation of the symptoms, each of which ranged from 0 to 9 points.

#### Suicidal ideations and suicide attempts

SIs and SAs are categorical variables that concern whether the adolescents had had recurring thoughts of taking their lives or had tried to take their lives during the 2 weeks before the survey. The response alternatives were ‘no’ (0) or ‘yes’ (1).

#### Subjective social status and subjective economic wealth

**Figure 1.**
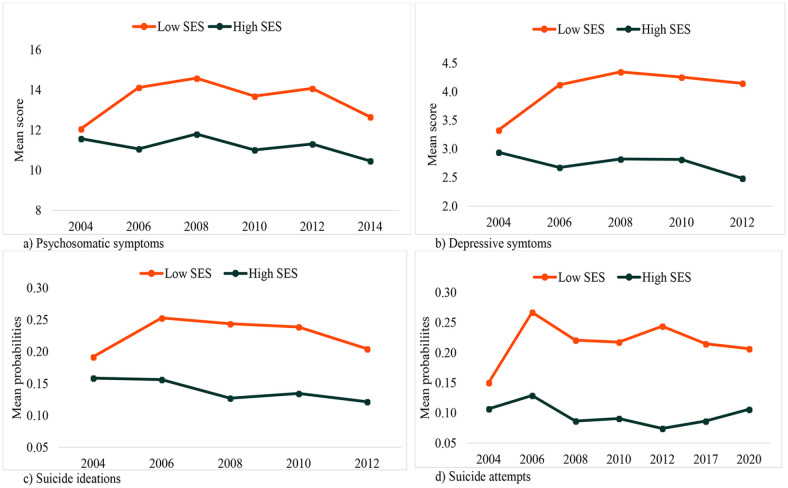
Distribution of mean scores of mental health problems by year of survey, at varying values of subjective social status and subjective economic wealth (socioeconomic status; SES): (a) psychosomatics symptoms; (b) depressive symptoms; (c) probabilities of suicidal ideations; (d) probabilities of suicide attempts.

To measure SES, we used a measure of subjective social status and subjective economic wealth [17, 23]. A seven-point Likert scale was used to assess the adolescents’ perception of their families’ SES with the following question: ‘Imagine society as a ladder. Families with the least money are at the bottom while those with the most money are at the top. If you think about your family’s wealth compared to that of society at large, where would you place your family on the ladder?’ Alternatives ranged from 0 (lowest) to 6 (highest). This variable is referred to as ‘SES’ throughout the document for ease of readability. The variable was treated as being on an interval scale in the regression equations, but to illustrate the distribution of the mean scores across SES gradients we dichotomised SES into low or medium/high with a cut-off point of −1 standard deviation (SD). Accordingly, all with SD of −1 or less were in the ‘low SES group’ and the rest were in the ‘high SES group’(see Figure 1).

#### Sex

Sex was the other independent variable, concerning whether the participant was a boy or a girl, and the responses were coded as either boy (0) or girl (1).

### Statistical analyses

**Figure 2.**
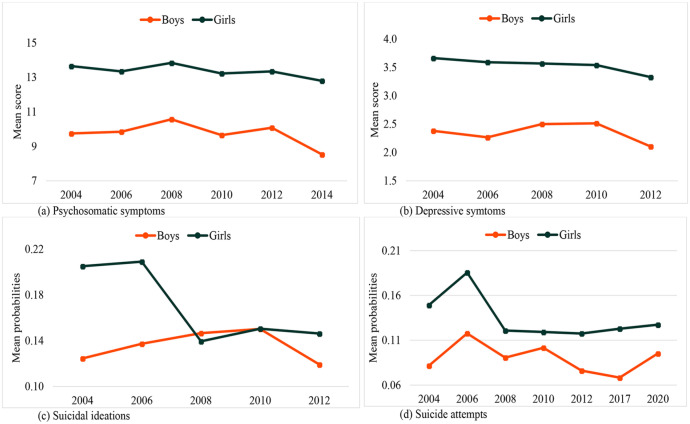
Distribution of mean scores of mental health problems by year of survey, at varying values of sex: (a) psychosomatics symptoms; (b) depressive symptoms; (c) probabilities of suicidal ideations; (d) probabilities of suicide attempts.

We employed linear regression to estimate the trends in PSSs and DSs by interpreting the survey-year’s coefficients. We used unstandardised beta coefficients in the estimation of trends, and we also carried out *z* transformation to standardise and make possible comparison between the estimation of trends in PSSs and DSs that were measured on different scales. For the categorical outcomes, SIs and SAs, we used logistic regression. To estimate the trends, we used odds ratios that were calculated from survey years’ coefficients. Each survey year was recoded to a natural number and treated as a continuous variable, except for the results presented in Figures 1 and 2, in which it was used as a data point that represents the specific year at which data were collected. To assess the role of SES or sex in moderating the trends in mental health problems, we used interaction terms between survey year and SES or sex. Two models (model 1 and model 2) were fitted for each indicator (dependent variable) and set of independent variables (year of survey, SES, and sex). The first model contained a dependent variable and the independent variables. In the second model, interaction terms between survey year and SES or sex, respectively, were added to assess the role of SES or sex in moderating the trend in mental health problems. R-squared change and χ^2^ change in the Omnibus test of model coefficients were used for linear and logistic regressions, respectively, to test the significance of differences between models 1 and 2. We used Cohen’s *d* [24] and the Agresti–Caffo (AC) confidence intervals [25] for measuring the effect sizes of mean differences for the continuous, and of proportional differences for the categorical, outcome variables, respectively. SPSS version 28 was used for the data analyses.

## Results

Mean values and SDs, and also the missing proportions of each outcome variable across the waves, are presented in Supplemental Table IV. The mean scores ranged from 10.7–12.2 for PSSs, 2.7–3.0 for DSs, 0.13–0.17 for SIs, and 0.10–0.15 for SAs. SAs have the largest proportion of missing values (9%), because the item was not considered during the 2014 wave.

### Trends in adolescent mental health problems

The results show decreasing trends over the years of the survey for all the indicators of adolescent mental health problems. We found significantly decreasing trends in PSSs (B = −0.131; confidence interval (CI) −0.182, −0.079), DSs (B = −0.046; CI −0.079, −0.013), SIs (odds ratio (OR) 0.933; CI 0.901, 0.967), and SAs (OR 0.960; CI 0.635, 0.985) (see model 1 in Table II and Table III and Supplemental Figure 3). *Z* transformation of the continuous outcome variables, PSSs and DSs, which were measured on different scales, shows that the trend in DSs declined faster than the trend in PSSs over time among those with high SES (Supplemental Table V). The coefficients show the changes in outcome that are associated with each survey year. The effect sizes of the standardised mean differences between the mean scores at the start and end of the study periods, as measured by Cohen’s *d* for PSSs (*d*=0.16; CI [0.11, 0.22]) and DSs (*d*=0.12, CI [0.06, 0.17]), were small. Similarly, small and non-significant effect sizes for SIs (AC 0.03, CI [0.01, 0.05]) and SAs (AC 0.001, CI [–0.06, 0.06]), respectively, were found for the differences between the proportions at the start and end of the study periods.

**Table II.** Linear regression results for the effect of year of survey on: (a) psychosomatic symptoms; and (b) depressive symptoms among adolescents.

Variables	Model 1			Model 2		
	B	95.0% CI for B		B	95.0% CI for B	
		Lower	Upper		Lower	Upper
Psychosomatic symptoms (a)^a^						
Year of survey	−0.131***	−0.182	−0.079	−0.114**	−0.188	−0.041
Family SES	−0.653***	−0.731	−0.576	−0.677***	−0.754	−0.599
Sex (girl)	3.517***	3.343	3.691	3.512***	3.338	3.686
SES–year interaction				−0.115***	−0.160	−0.070
Sex–year interaction				−0.003	−0.107	0.0100
R^2^ change = 0.001; F(2, 15121) = 12.68; *P*<0.001						
Depressive symptoms (b)^b^						
Year of survey	−0.046**	−0.079	−0.013	−0.003	−0.050	0.043
Family SES	−0.322***	−0.362	−0.282	−0.341***	−.382	−0.300
Sex (girl)	1.132***	1.041	1.223	1.121***	1.030	1.212
SES-year interaction				−0.084***	−0.114	−0.055
Sex-year interaction				−0.073*	−0.138	−0.007

R^2^ change = 0.002; F(2, 13132) = 17.58; *P*<0.001.

*** *P*<0.001; **P<0.01; *P<0.05.

B: unstandardised B coefficient; CI: confidence interval; SES: subjective social status and subjective economic wealth.

a The measurement scale ranged from 0 to 32 for psychosomatic symptoms.

b From 0 to 9 for depressive symptoms.

**Table III.** Logistic regression results for the effects of year of survey on: (c) suicidal ideations; and (d) suicide attempts among adolescents.

Variables	Model 1			Model 2		
	OR	95% CI for OR		OR	95% CI for OR	
		Lower	Upper		Lower	Upper
Suicidal ideations (c)						
Year of survey	0.933***	0.901	0.967	1.013	0.961	1.067
High vs. low SES	0.869***	0.833	0.908	0.857***	0.820	0.895
Girls vs. boys	1.288***	1.168	1.419	1.258***	1.140	1.387
SES–year interaction				0.953**	0.924	0.983
Sex–year interaction				.855***	0.797	0.918
χ^2^(2)_(χ2model2 – χ2model1)_ = 26.05; *P*<0.001						
Suicide attempts (d)						
Year of survey	0.960**	0.935	0.985	0.981	0.941	1.021
High vs. low SES	0.789***	0.755	0.825	0.781***	0.746	0.818
Girls vs. boys	1.549***	1.399	1.715	1.528***	1.378	1.695
SES–year interaction				0.979	0.957	1.002
Sex–year interaction				0.962	0.913	1.014

χ^2^(2)_(χ2model2 – χ2model1)_ = 5.22; *P*=0.074.

*** *P*<0.001; **P<0.01; *P<0.05.

OR: odds ratio; CI: confidence interval; SES: subjective social status and subjective economic wealth.

### SES and trends in mental health problems

In this study, although mental health problems showed declining trends over time, the rate of decline varied across gradients of SES, as shown in Figure 1, and confirmed by significant interaction effects (survey year × SES) in all the domains except for SAs (see Table II(a) and (b) and Table III(c) and (d)). Trends in all indicators of mental health problems, except for SAs, increased over time among adolescents with low subjective SES, but decreased among those with high subjective SES (PSSs, B = −0.115; CI −0.160, −0.070; DSs, B = −0.084; CI −0.114, −0.055; and SIs, OR 0.953; CI 0.924–0.983).

### Sex and trends in mental health problems

Girls consistently reported significantly higher burdens of mental health problems than boys throughout the survey years. Trends in only two of the four indicators were moderated by sex, as confirmed by the significant interaction effects (survey year × sex) shown in Table II(b) and Table III(c). Sex differences emerged for DS (B = −0.073; CI −0.138, −0.007) and SIs (OR 0.855; CI 0.797, 0.918), in which the declines were more pronounced among girls than boys, see Figure 2(a)–(d). There was no significant difference between boys and girls regarding trends in PSSs and SAs.

## Discussion

This study investigated time trends in PSSs (2004–2014), DSs (2004–2012), SIs (2004–2012) and SAs (2004–2020) among 15-year-olds in the Västmanland region in Sweden. We also looked at whether the trends were moderated by adolescents’ subjective SES and sex. Overall, we found decreasing trends in adolescent mental health problems over time, but the trends varied with both SES, measured as subjective social status and subjective economic wealth, and sex.

The first striking finding of the study is that trends in adolescent mental health problems have slightly decreased over time, but with generally small effect sizes. This contrasts with findings of previous studies of the same population that reported increasing trends [1–3]. The difference between the studies can be due to the different time points considered. For example, our study used data collected at more time points, which makes it difficult to make a comparison with the study of Blomqvist et al. [1], which used data from only two time points. These are too few [5] and far apart from each other [26] to estimate change adequately over time. Also, a difference in time points can explain the differing findings between Bremberg [2], which covered the period 1990–2010, and this study, which focused on 2004–2020. We found that most indicators started declining after 2008 (see Supplemental Figure 3), which is consistent with a study in Denmark that reported depression among adolescents peaking in 2010 but declining thereafter [27], and also with Högberg et al. [3], in which the trends in PSSs either declined or started to level off in 2005 (see Figure 2 in the Högberg study). Taken together, all these studies, including ours, suggest a decline in youth mental health until 2005–2010, followed by a phase of stabilisation/improvement after that period.

Nevertheless, the decreasing trends in mental health problems were not equally spread across groups. The trends have significantly decreased among adolescents with high subjective social status and subjective economic wealth, but increased among those with low subjective social status and subjective economic wealth. We know from previous studies that there is a strong association between subjective SES and mental health problems attributable to resentment, dissatisfaction, or worry resulting from perceived relative socioeconomic position [18]. The current study, however, brings another dimension to the association, in which trends in mental health problems over time are dependent on changes in subjective SES, defined here as subjective social status and subjective economic wealth. A possible explanation for the association is that SES moderates the effects of other predictors of mental health, such as social and school-related factors. For instance, research shows that students with low SES have greater problems at school, which in turn are associated with more mental health problems [28]. In Sweden, following the school reform of the 1990s, differences in students’ performances between schools have been attributed to increasing disparities between both municipalities and schools in terms of the resources available to invest in education [29]. This indicates the need to consider overall structural contexts in addition to individual-level factors, as in Högberg [30], to understand better the complex relationship between school-related factors and mental health problems. Future studies might need to consider such societal changes to disentangle these complexities.

The other notable finding of this study lies in the differences found in the rates of decline between boys and girls in some of the trends. Although girls reported a greater burden of mental health problems than boys throughout the study, the declines in the trends in DSs and SIs were significant among girls but not among boys. While the different burdens of mental health problems on girls and boys might be explained by the findings of previous studies [19], the reasons behind the sex differences in the trends need further investigation. Future studies should investigate whether and/or why the factors that have contributed to overall declining trends might have had larger effects on the trends among girls.

This study has some limitations. First, data were not uniformly available for all the mental health indicators for the whole period of 2004–2020. As a result, we conducted separate analyses for each mental health problem for the periods data were available. Nevertheless, our study is one of the few that has analysed trends in four indicators in a single study. We believe that this could help overcome the challenge of trying to explain complex issues such as mental health problems [9] using a single indicator, whereas the use of multiple indicators may better illustrate the overall picture [13]. It should also be noted that the study used a repeated cross-sectional design, which may not be suitable for drawing causal conclusions. Nonetheless, a previous study has shown that repeated cross-sectional studies can adequately estimate population changes over time [6]. As school data were not available, the effects of inter-school differences were not accounted for, and future studies might need to investigate the effects these differences may have on the trends in mental health problems. Finally, the study was based on data from the Västmanland region in Sweden, excluding children in special schools, which might limit the generalisability of the results even to Swedish adolescents. Nevertheless, the results might still be relevant to the whole country as the Västmanland region is considered representative of the country’s population, based on various parameters such as the distributions of education, employment, income, and urban and rural settings [21].

## Conclusions

This study shows that the mental health of 15-year-olds in Västmanland improved from 2004 to 2020. However, this does not hold true for all groups of young people: the trends have significantly decreased over time among girls and youth with high subjective SES, measured as subjective social status and subjective economic wealth, and increased among those with low SES, but they did not change among boys. This study confirms that socioeconomic inequalities are associated with inequalities in health impacts, including on mental health [17], and sheds some light on growing health inequalities in the population. The possible disparities between trends in mental health development should be looked at closely over the coming years.

## Supplemental Material

sj-docx-1-sjp-10.1177_14034948231165552 – Supplemental material for Trends in adolescent mental health problems 2004–2020: do sex and socioeconomic status play any role? Supplemental material, sj-docx-1-sjp-10.1177_14034948231165552 for Trends in adolescent mental health problems 2004–2020: do sex and socioeconomic status play any role? by Benti Geleta Buli, Peter Larm, Kent Nilsson and Fabrizia Giannotta in Scandinavian Journal of Public Health

sj-docx-2-sjp-10.1177_14034948231165552 – Supplemental material for Trends in adolescent mental health problems 2004–2020: do sex and socioeconomic status play any role? Supplemental material, sj-docx-2-sjp-10.1177_14034948231165552 for Trends in adolescent mental health problems 2004–2020: do sex and socioeconomic status play any role? by Benti Geleta Buli, Peter Larm, Kent Nilsson and Fabrizia Giannotta in Scandinavian Journal of Public Health

sj-docx-3-sjp-10.1177_14034948231165552 – Supplemental material for Trends in adolescent mental health problems 2004–2020: do sex and socioeconomic status play any role? Supplemental material, sj-docx-3-sjp-10.1177_14034948231165552 for Trends in adolescent mental health problems 2004–2020: do sex and socioeconomic status play any role? by Benti Geleta Buli, Peter Larm, Kent Nilsson and Fabrizia Giannotta in Scandinavian Journal of Public Health
