# Supplementary material for: Trends in adolescent mental health problems 2004–2020: Do sex and socioeconomic status play any role?
Source: Scand J Public Health. 2023 May 4;52(5):565–72. doi: 10.1177/14034948231165552 (PMC11292962; doi:10.1177/14034948231165552)
Supplement: sj-docx-1-sjp-10.1177_14034948231165552 – Supplemental material for Trends in adolescent mental health problems 2004–2020: do sex and socioeconomic status play any role? [file sj-docx-1-sjp-10.1177_14034948231165552.docx]

Figure 3. Trends of mean scores of mental health problems by year of survey: (a) psychosomatic symptoms, (b) depressive symptoms, (c) suicidal ideations, and (d) suicide attempts reported by adolescents aged 15 years
