# Supplementary material for: Trends in adolescent mental health problems 2004–2020: Do sex and socioeconomic status play any role?
Source: Scand J Public Health. 2023 May 4;52(5):565–72. doi: 10.1177/14034948231165552 (PMC11292962; doi:10.1177/14034948231165552)
Supplement: sj-docx-2-sjp-10.1177_14034948231165552 – Supplemental material for Trends in adolescent mental health problems 2004–2020: do sex and socioeconomic status play any role? [file sj-docx-2-sjp-10.1177_14034948231165552.docx]

Table 4. Mean scores of outcome variables, their standard deviations, and proportion of missing values

| **Year of survey** | **PSS** | **DS** | **SI** | **SA** |
| --- | --- | --- | --- | --- |
|  | ***Mean (S.D)*** | ***Mean (S.D)*** | ***Mean (S.D)*** | ***Mean (S.D)*** |
| 2004 | 11.7 (5.78) | 3.0 (2.63) | 0.17 (0.37) | 0.12 (0.32) |
| 2006 | 11.6 (5.82) | 2.9 (2.80) | 0.17 (0.38) | 0.15 (0.36) |
| 2008 | 12.2 (5.83) | 3.0 (2.76) | 0.14 (0.35) | 0.11 (0.31) |
| 2010 | 11.4 (5.79) | 3.0 (2.78) | 0.15 (0.36) | 0.11 (0.31) |
| 2012 | 11.7 (5.69) | 2.7 (2.71) | 0.13 (0.34) | 0.10 (0.30) |
| 2014 | 10.7 (5.81) |  |  | DNA |
| 2017 |  |  |  | 0.10 (0.30) |
| 2020 |  |  |  | 0.11 (0.32) |
| Overall | 11.57 (5.81) | 2.9 (2.74) | 0.15 (0.36) | 0.12 (0.32) |
| Missing (%) | 1.4 | 0.0 | 4.2 | 9.0 |
| PSS =psychosomatic symptoms, DS=depressive symptoms, SI=suicidal ideations, SA=Suicidal attempts, DNA=Data not available | | | | |
