# Supplementary material for: Trends in adolescent mental health problems 2004–2020: Do sex and socioeconomic status play any role?
Source: Scand J Public Health. 2023 May 4;52(5):565–72. doi: 10.1177/14034948231165552 (PMC11292962; doi:10.1177/14034948231165552)
Supplement: sj-docx-3-sjp-10.1177_14034948231165552 – Supplemental material for Trends in adolescent mental health problems 2004–2020: do sex and socioeconomic status play any role? [file sj-docx-3-sjp-10.1177_14034948231165552.docx]

Table 5. Linear regression results of the effect of year of survey, adjusted for sex and SES, on: a) psychosomatic symptoms, and b) depressive symptoms among adolescents after z-transformation of the dependent variables

| **Variables** | **Model 1** | | | **Model 2** | | |
| --- | --- | --- | --- | --- | --- | --- |
|  | B | 95.0% CI for B | | B | 95.0% CI for B | |
|  |  | Lower | Upper |  | Lower | Upper |
| **Psychosomatic symptoms (a)** | | | | | | |
| Year of Survey | -.023*** | -.031 | -.014 | -.020** | -.032 | -.007 |
| Family SES | -.113*** | -.126 | -.099 | -.117*** | -.130 | -.103 |
| Sex (girl) | .606*** | .576 | .636 | .605*** | .575 | .635 |
| SES-year interaction |  |  |  | -.020*** | -.028 | -.012 |
| Sex-year interaction |  |  |  | -.001 | -.018 | .017 |
| *R^2^-change = .001; F(2, 15121) = 12.68; p<.001* | | | | | | |
| **Depressive symptoms (b)** | | | | | | |
| Year of Survey | -.017** | -.029 | -.005 | -.001 | -.018 | .016 |
| Family SES | -.117*** | -.132 | -.103 | -.124*** | -.139 | -.109 |
| Sex (girl) | .413*** | .380 | .446 | .409*** | .376 | .442 |
| SES-year interaction |  |  |  | -.031*** | -.041 | -.020 |
| Sex-year interaction |  |  |  | -.026* | -.051 | -.002 |
| *R^2^-change = .002; F(2, 13132) = 17.58; p<.001* | | | | | | |

*** p <0.001, ** p <0.01, * p <0.05; B = unstandardized b-coefficient, CI = Confidence interval, SES = subjective social status and subjective economic wealth

^(†)^ The measurement scale ranged from 0 to 32for psychosomatic symptoms, and from 0 to 9 for ^(††)^ depressive symptoms.
